# Angular correlations of photons from solution diffraction at a free-electron laser encode molecular structure

**DOI:** 10.1107/S2052252516013956

**Published:** 2016-09-26

**Authors:** Derek Mendez, Herschel Watkins, Shenglan Qiao, Kevin S. Raines, Thomas J. Lane, Gundolf Schenk, Garrett Nelson, Ganesh Subramanian, Kensuke Tono, Yasumasa Joti, Makina Yabashi, Daniel Ratner, Sebastian Doniach

**Affiliations:** a Department of Applied Physics, Stanford University, Stanford, CA 94305, USA; b Department of Physics, Stanford University, Stanford, CA 94305, USA; c SLAC National Accelerator Laboratory, Menlo Park, CA 94025 USA; d Department of Physics, Arizona State University, Tempe, AZ 85287, USA; e Japan Synchrotron Radiation Research Institute (JASRI), Kouto 1-1-1, Sayo, Hyogo 679-5198, Japan; f RIKEN SPring-8 Center, Kouto 1-1-1, Sayo, Hyogo 679-5148, Japan

**Keywords:** angular photon correlations, solution diffraction, XFELs, correlated X-ray scattering, gold nanoparticles

## Abstract

During X-ray exposure of a molecular solution, photons scattered from the same molecule are correlated. If molecular motion is insignificant during exposure, then differences in momentum transfer between correlated photons are direct measurements of the molecular structure. In conventional small- and wide-angle solution scattering, photon correlations are ignored. This report presents advances in a new biomolecular structural analysis technique, correlated X-ray scattering (CXS), which uses angular intensity correlations to recover hidden structural details from molecules in solution. Due to its intense rapid pulses, an X-ray free electron laser (XFEL) is an excellent tool for CXS experiments. A protocol is outlined for analysis of a CXS data set comprising a total of half a million X-ray exposures of solutions of small gold nanoparticles recorded at the Spring-8 Ångström Compact XFEL facility (SACLA). From the scattered intensities and their correlations, two populations of nanoparticle domains within the solution are distinguished: small twinned, and large probably non-twinned domains. It is shown analytically how, in a solution measurement, twinning information is only accessible *via* intensity correlations, demonstrating how CXS reveals atomic-level information from a disordered solution of like molecules.

## Abstract 2

An atomic twinning structure is observed by averaging intensity correlations from many snapshots of gold nanoparticles in solution.

## 1. Introduction   

Correlated X-ray scattering (CXS), also referred to as fluctuation X-ray scattering, is an emerging field which involves using angular intensity correlations to recover the average local structure of molecules in a random ensemble (Kam, 1977 ▸). In a solution exposure, molecules in random orientations scatter photons in all directions. Two photons scattered from the same molecule are correlated *via* their mutual momentum-transfer dependence on the molecular structure. As such, the difference in momentum transfer between two correlated photons is a measure of the molecular structure. However, this signal is submerged in intrinsic noise on a per-exposure basis due to the uncorrelated scattering from the large number of molecules in solution. In order to extract this structural information, one can average angular intensity correlations for many exposures of the solution in different orientational ensembles. If the molecules move during exposure, the momentum transfer differences between correlated photons will become less clearly defined, so to maximize the signal-to-noise ratio it is advantageous to use rapid exposures. At the Spring-8 Ångström Compact XFEL facility (SACLA), the X-ray free-electron laser (XFEL) pulse duration is about 100 fs, much faster than the rotational diffusion timescales of a typical molecule in solution. With a pulse repetition rate that can be tuned to 30 Hz, SACLA provides an ideal experimental setup for recording intensity correlations.

Solution CXS measurements at an XFEL have the potential to reveal the internal structural details of proteins and other biomolecules without the use of crystallization (Saldin, Poon *et al.*, 2010 ▸; Saldin, Shneerson *et al.*, 2010 ▸; Saldin *et al.*, 2009 ▸; Pande *et al.*, 2014 ▸; Schenk *et al.*, 2015 ▸), although recovering the intensity correlations from solution diffraction measurements is challenging. In order to use CXS effectively on solution data, it is necessary to develop a robust analysis technique that can effectively extract intensity correlations while minimizing systematic noise on a per-shot basis. To this end, we present a detailed description of a solution CXS experiment done at SACLA based on small gold nanoparticles (NPs). We selected gold NPs due to their large atomic scattering cross section. Experimental work testing CXS has been published on iron oxide nano-rice samples (Liu *et al.*, 2013 ▸) and lithographically generated dumb-bells (Chen *et al.*, 2012 ▸). These experiments used relatively low-angle scattering data, with one or a few exposed molecules per exposure. Here, we present measurements on three-dimensional solutions of tens of thousands of gold NPs measured at wide scattering angles.

NP suspensions are used in chemical catalysis, and their chemical properties are directly related to their overall shape and atomic structure (Yacamán *et al.*, 1981 ▸; Narayanan & El-Sayed, 2005 ▸, 2004 ▸). Past work describing the thermodynamics and kinetics of NP growth and formation (Ino, 1969 ▸; Marks, 1983 ▸, 1984 ▸; Howie & Marks, 1984 ▸; Ringe *et al.*, 2013 ▸) has revealed that smaller NPs tend to form complicated twinning structures, *e.g.* decahedral and icosahedral twins (Heinemann *et al.*, 1979 ▸; Yacamán *et al.*, 1979 ▸; Langille *et al.*, 2012 ▸; Yang, 1979 ▸; Yang *et al.*, 1979 ▸; Dai *et al.*, 2002 ▸). Conventional powder X-ray diffraction measurements (small- and wide-angle scattering), used widely in industry to characterize ensembles of NPs, are isotropic averages and cannot show signs of twinning. Traditionally, twinning has been observed using electron microscopy and electron tomography (Marks & Smith, 1981 ▸; Yacamán & Avalos-Borja, 1992 ▸; Chen *et al.*, 2013 ▸), where one images single NP projections, but this is only possible due to the stability of heavy-atom nanocrystalline structures.

In general, soft-matter biomolecules cannot withstand high dose rates of electron or X-ray exposure, leading to radiation damage. Using the ‘diffract before destroy’ property of XFEL measurements, one can measure correlated photons arising from intense exposure of a solution sample before the sample undergoes damage. In such cases, CXS is unique in the amount of structural information it can recover from correlated photons. CXS has been extensively explored as a tool to investigate two-dimensional systems (Kurta, Ostrovskii *et al.*, 2013 ▸; Schroer *et al.*, 2014 ▸; Lehmkühler *et al.*, 2014 ▸; Kurta *et al.*, 2012 ▸; Pedrini *et al.*, 2013 ▸; Saldin, Poon, Bogan *et al.*, 2011 ▸). However, in three-dimensional systems the structural information encoded in the data becomes more difficult to extract using CXS techniques (Elser, 2011 ▸). If one or a few three-dimensional objects are exposed during each exposure, then one can use symmetry arguments to recover structural information content (Kam, 1980 ▸; Poon & Saldin, 2015 ▸; Chen *et al.*, 2012 ▸; Liu *et al.*, 2013 ▸; Starodub *et al.*, 2012 ▸; Saldin, Poon, Schwander *et al.*, 2011 ▸). When the number of exposed three-dimensional objects increases, one can use the correlated intensities to infer local structural characteristics (Wochner *et al.*, 2009 ▸; Altarelli *et al.*, 2010 ▸; Kurta, Chesnokov *et al.*, 2013 ▸; Malmerberg *et al.*, 2015 ▸), to resolve structural changes (Pande *et al.*, 2015 ▸) and, potentially, to refine atomic models in an iterative procedure (Liu *et al.*, 2012 ▸). In this paper we report on CXS as a tool to investigate a three-dimensional ensemble of gold NPs, where each exposure is from samples composed of many NPs. We will show how CXS reveals NP twinning from solution scattering measurements recorded at an XFEL, and how this otherwise hidden information may be extracted by correlating the scattered intensities.

## 2. Experimental   

### 2.1. Background   

**Figure 1:**
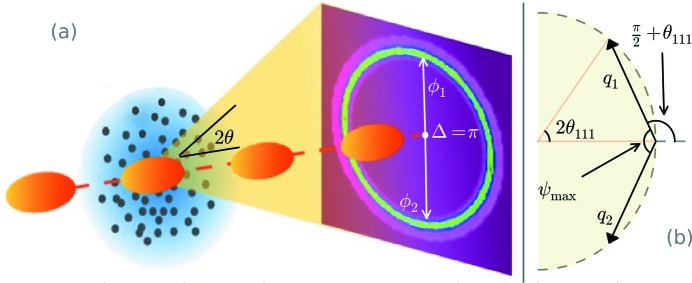
Schematic diagrams of the experimental setup and geometry. (*a*) X-ray pulses (orange) exposing a solution of gold nanoparticles. Shown in bright green is the {111} Bragg ring. Also shown are two positions along the Bragg ring, 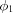 and 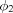, separated by an angle Δ = π. Artwork courtesy of Gregory M. Stewart (SLAC). (*b*) The elastic scattering geometry corresponding to the case when Δ = π. Note that ψ_max_ < π at wide angles.

**Figure 2:**
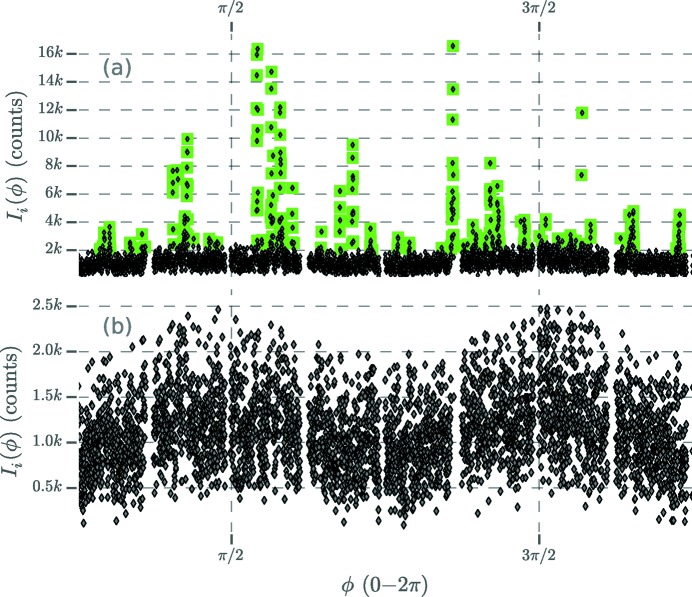
Separation of bright Bragg spots in the angular intensity profile. (*a*) The {111} Bragg ring intensity of a single snapshot exposure *i*. Highlighted in green are the brightest intensities. (*b*) The same as (*a*), but the bright Bragg spots are removed, leaving behind the moderate intensity, which forms a relatively noisy signal. The angular gaps in (*a*) and (*b*) represent gaps between the detector pixel panels. The variation in counts periodic in π is due to beam polarization. Other non-uniformities occur in the analysis, including detector shadows (Fig. S4 in the supporting information). We correlate the bright and moderate intensities separately (the results are shown in Fig. 3 ▸).

An object in solution exposed to sufficient X-ray flux can scatter photons into at least two directions, **q**_1_ and **q**_2_. While the orientation of this object can be random, the angle defined by **q**_1_ and **q**_2_

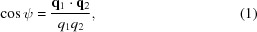
is not; it is determined by the object’s internal atomic structure. A crystalline NP scatters photons into discrete Bragg vectors **q**_*hkl*_. We define a detector whose pixels correspond to a set of Bragg vectors {**q**}. Let 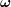 be a triple of Euler angles defining an NP orientation relative to some axis (*e.g.* that of an X-ray beam). An NP at orientation 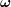 can scatter photons into the detector, provided 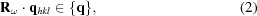
where 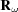 is an operator which rotates the NP from some pre-defined arbitrary orientation into 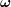. We assume that a small fraction of NPs in solution are oriented such that condition (2) is met for two Bragg vectors, 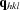 and 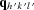, *i.e.* a small fraction of NPs are oriented such that they can produce two Bragg reflections on the detector. The NPs thus oriented that scatter photons into both 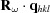 and 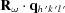 will produce intensity correlations between pairs of Bragg vectors in {**q**} whose angular separation ψ is defined by 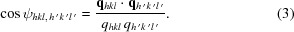
The angle 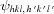 is also the interplanar angle between crystallographic planes *hkl* and 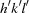. Typically, the pixels {**q**} are arranged on a planar detector, assumed to be perpendicular to the forward X-ray beam (Fig. 1 ▸ *a*). With such a setup, it is often convenient to calculate correlations in terms of the azimuthal angle 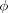 which spans the detector plane 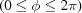. The azimuthal degree of separation, Δ = 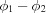, between any two pixels on the detector can be expressed in terms of cosψ *via*

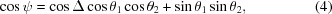
(Fig. 1 ▸ *b*), where θ is half the Bragg angle for elastically scattered photons at wavelength λ, defined by 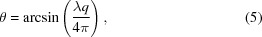
(Fig. 1 ▸ *a*). Geometrically, ψ has a maximum when Δ = π, hence 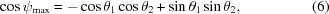
which sets a bound on the correlation angles ψ that can be measured in a given experiment. Therefore, by increasing the energy of the beam (lowering λ and hence θ), one can measure a wider range of correlation angles ψ. Note that, at small scattering angles, 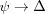 (Fig. 1 ▸ *b*). Recently published CXS experiments have been conducted in this small-angle limit, with one exception being our past work done on a microfocus synchrotron radiation beamline (Mendez *et al.*, 2014 ▸). For the current experiment, we calculated correlations along the {111} Bragg ring 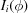 for each exposure *i* (Fig. 2 ▸). Angular correlations were computed in the azimuthal component of the detector 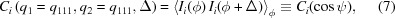
and the signal was expressed in terms of 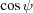 using equation (4). The low order, anisotropic profile in 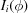 will give rise to strong artifactual correlations that are independent of the molecular structure in the sample. Rather than summing the correlations 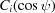, we instead subtract pairs of exposures similar in their anistropies as determined by a 15th degree Chebyshev polynomial fit, and then correlate the differences. For details regarding the fits, see section S1.6. This method of using subtraction to suppress artifactual CXS signal was first conceptualized by Kam *et al.* (1981 ▸). We define the difference correlation 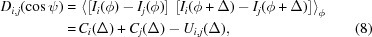
where *U_i,j_*(Δ) = 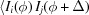 + 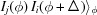 represents any artifactual signal. In practice, residual artifactual correlations can still be observed in the average difference correlation (for an example, see Fig. S6 in the supporting information). Successful application of CXS data to structural studies depends on one’s ability to distinguish the scattered photon correlations from artifactual signals. To this end, we employ a Friedel symmetry constraint. Friedel’s law states that *I*(**q**) = *I*(−**q**) (in the absence of anomalous scattering). Hence, if one measures a physical correlation at an angle ψ = 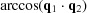, one should measure the same correlation at an angle π − ψ = 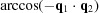. This implies that a pure CXS function should be mirror-symmetric about ψ = π/2 (cosψ = 0). Any signal violating this symmetry is likely artifactual. We define the Friedel difference correlation 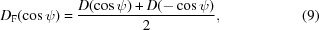
which enhances the true CXS information while minimizing false correlation peaks that defy Friedel symmetry.

In a typical exposure, a fraction of NPs are oriented such that they scatter into the detector, hence an even smaller fraction will be oriented such that they scatter into multiple detectable directions (Mendez *et al.*, 2014 ▸). Therefore, the average exposure includes a large fraction of randomly scattered and uncorrelated photons (owing to the orientation randomness in a solution). While the CXS signal-to-noise ratio for a single exposure is much less than unity, the ratio scales with *N* ^1/2^, the square root of the number of averaged exposures (Kirian *et al.*, 2011 ▸). We consider an exposure to be a snapshot, meaning the NPs should not be moving significantly throughout the exposure duration. This is guaranteed by the femtosecond timescale pulses of the SACLA facility (Neutze *et al.*, 2000 ▸). CXS can also be conducted at synchrotron radiation facilities, provided that the samples are prepared in an antifreeze suspension and cooled during exposure to prevent motion of the particles (Mendez *et al.*, 2014 ▸; Kam *et al.*, 1981 ▸).

### 2.2. Sample preparation and experimental setup   

Water-soluble gold NPs (specified to be 60 nm in diameter) were purchased from Nanopartz Inc. (Loveland, Colorado, USA) at a concentration of 100 mg ml^−1^. The solution reportedly contained 5.21 × 10^13^ NPs ml^−1^, with fewer than 0.01% of NPs less than 20 nm in diameter, although the exact details of the manufacturer’s sample characterization could not be provided at the time of inquiry. It is worth noting that our sample preparation protocol could have altered these numbers. Prior to exposure, the gold NPs were suspended in a lipid cubic phase (LCP) buffer. A mixture of 40% NP suspension and 60% toluene was emulsified by passing the solution back and forth through a 250 µm aperture between two syringes according to an established protocol for preparing LCP (Caffrey & Cherezov, 2009 ▸). The final concentration of the gold–LCP solution was 40 mg ml^−1^. A Hamilton 7780-01 syringe needle with inner diameter 130 µm was attached to one of the LCP syringes, which was then installed in a purpose-built injector which used a remotely controlled step motor to drive the syringe plunger at variable speeds. The injector speed was optimized to ensure a good-quality flow of the gold–LCP emulsion into the X-ray laser beam. A minimum plunger speed was set to ensure a lateral flow rate of 90 µm s^−1^ so that the solution was sufficiently exchanged between XFEL pulses. The SACLA beam energy was set to 8.6 keV (λ = 1.442 Å) and focused down to a spot size of roughly 1.5 × 2.4 µm. Given an exposed sample volume of 1.5 × 2.4 × 130 µm^3^ and a dilution factor of 0.4, we estimate that there were roughly 9.8 × 10^3^ NPs illuminated during each exposure. The beam pulse repetition rate was 30 Hz. The scattered photons were measured using an MPCCD eight-panel detector in a wide-angle setup, capable of probing momentum transfer up to 3.4 Å^−1^. The scattering angle θ_111_ was 17.83° and, for {111} autocorrelations [θ_1_ = θ_2_ = θ_111_ in equation (6)], ψ_max_ was 144.3°. With this setup we acquired roughly 5 × 10^5^ snapshot exposures of gold NPs. As previously reported, straightforward computation of equation (7) is dominated by artifactual correlations associated with the experiment (Mendez *et al.*, 2014 ▸). Examples of these correlations include pixel cross-talk, detector shadows and scattering anisotropies due to an inhomogeneous sample. Assuming that different exposures will have similar artifactual asymmetries, equation (8) will suppress any asymmetries *via* subtraction, thus minimizing any artifactual correlation signal.

## 3. Results   

### 3.1. Data analysis   

Prior to correlation, we separated the {111} Bragg ring intensity 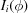 into two components: the brightest Bragg spots (Fig. 2 ▸ *a*) and the moderate intensities (Fig. 2 ▸ *b*). Specifically, we split the intensity according to 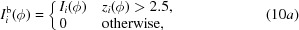


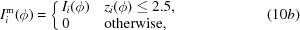
where 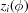 is a modified standard score in units of the median intensity around the Bragg ring (see Appendix *A* [App appa] for details).

**Figure 3:**
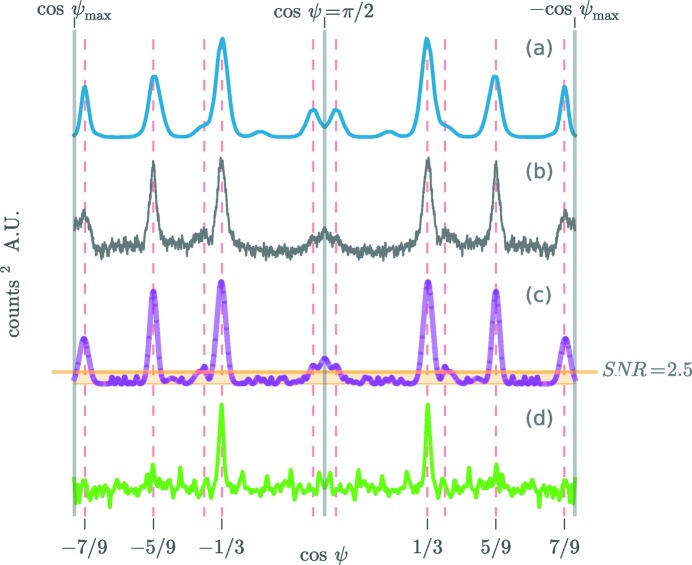
Simulated and measured angular correlation profiles of the {111} Bragg ring. (*a*) Simulated CXS for the gold decahedron in Fig. 5 ▸(*b*). For details of the simulation see section S2 in the supporting information. (*b*) The mirror-symmetric difference correlation of the moderate intensities, 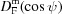, which imposes Friedel symmetry. These data represent an average of 1.6 × 10^5^ exposures. (*c*) The Gaussian fit *G*(cosψ) (Appendix *C* [App appc]) fit directly to 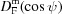. The horizontal line marks an SNR (Appendix *D* [App appd]) value of 2.5. There are many small peaks with a low SNR which are likely noise. (*d*) The mirror-symmetric difference correlation of the bright Bragg intensities, 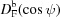. The absence of pronounced peaks at cosψ = 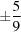 and 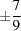 indicates that this signal possibly arises from a population of non-twinned scattering domains. Also, the relatively sharp width of the CXS peaks at cosψ = 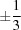 indicates that the corresponding NP domains are larger than the twinned domains which produced the CXS shown in part (*b*). Vertical dashed lines (red) are the predicted CXS signal from the NNT model, as well as other significant CXS signals.

We averaged the angular autocorrelation 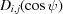 separately for the two clusters of intensities to resolve the CXS signals. The angular correlation of the moderate intensities, 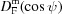, showed peaks at cosψ = 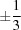, 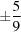 and 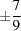, indicating the presence of twinning (Fig. 3 ▸ *b*; see *Discussion* for details). On the other hand, the CXS of the bright Bragg spots, 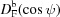, only showed peaks at cosψ = 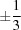 (Fig. 3 ▸ *d*), implying that the domains which scattered the brightest Bragg spots were most likely not twinned. This is to be expected, as NPs undergo stress-induced structural changes as they increase in size, creating a less ordered internal structure (Yacamán *et al.*, 2001 ▸) that might diminish the inter-domain correlations.

In a similar manner to how the width of a Bragg spot (peak) relates to the corresponding NP domain size, the width of the CXS peak can be used to infer the sizes of the NP domains which scatter correlated photons (Appendix *B*1[App appb]). We examine the full width at half-maximum (FWHM) of the CXS peaks at cosψ = 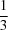 and find that the peak in 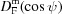 has a FWHM of 0.036 rad, while the peak in 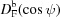 has a FWHM of 0.019 rad (Appendix *B*2[App appb]). Because the peak width is inversely proportional to the domain size, we infer that the bright Bragg spots come from larger NP domains within the population. From analysis of the CXS peak width (under the assumption that the NP domains are tetrahedra), we infer that the small twinned domains are tetrahedra of side length ≥12 nm, and the large domains are tetrahedra of side length ≥21 nm with a mean side length of 46 nm (Appendices *B*1[App appb] and *B*2[App appb]). To estimate the fraction of our sample which was small twinned domains, we considered the summed moderate intensity relative to the summed total intensity around each Bragg ring, averaged over exposures
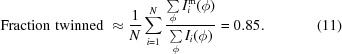
While we consider this estimate to be a rough approximation, populations of small (2–4 nm) thiol-capped gold NPs have been shown to obey similar distributions (Zanchet *et al.*, 2000 ▸), and these results may be extended to groups of larger NPs under certain growth conditions (Casillas *et al.*, 2012 ▸).

### 3.2. Data fitting and signal-to-noise ratio   

**Figure 4:**
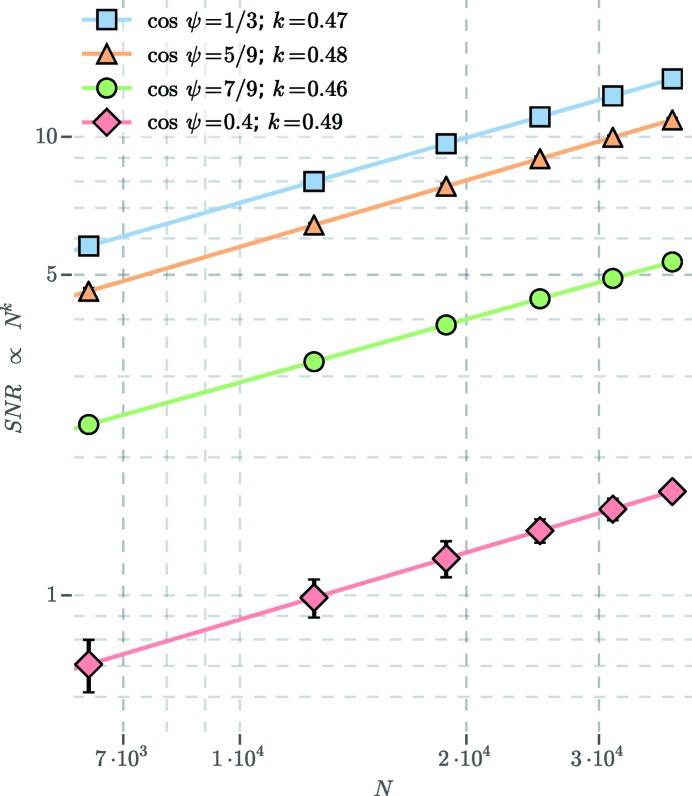
Signal-to-noise scaling. The estimated SNR of four significant CXS peaks in 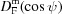 are plotted as a function of *N*, the number of averaged snapshot exposures. The SNR is defined in Appendix *D* [App appd]. The error bar shown is one standard deviation of the measurement.

**Figure 5:**
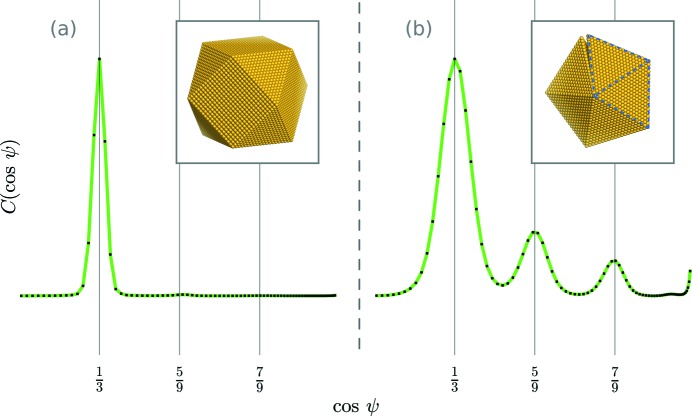
CXS of the {111} Bragg ring simulated for single- and multi-domain NP models. (*a*) The simulated CXS for a non-twinned cuboctahedron gold NP atomic model (section S2 in the supporting information). Note that, for single-domain gold particles, one would only expect a CXS signal at cosψ = 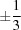, corresponding to the {111} interplanar angles of an f.c.c. crystal. We observed this CXS signal from the large domains in our sample. (*b*) The simulated CXS for a nearest-neighbor tetrahedron (NNT, outlined in dashed blue). Multi-twinned particles, such as the decahedron shown here, are composed of several NNT units. The angular gap in the decahedron results because the tetrahedra are each close-packed f.c.c. domains (Yang, 1979 ▸). The twinning gives rise to additional CXS peaks. We observed this signal from the small twinned NP domains.

For a more detailed description of the data-fitting procedure and computation of the signal-to-noise ratio, see Appendices *C* [App appc] and *D* [App appd], respectively. Fig. 3 ▸(*c*) shows the result of fitting a sum of Gaussians to 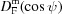 [for a description of the fitted function, see equation (32)]. The Gaussian amplitudes were used to compute the signal-to-noise ratio (SNR) of the CXS peaks. Fig. 4 ▸ shows the SNR scaling of four significant CXS peaks in 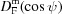. As expected (Kirian *et al.*, 2011 ▸), the SNR increases with the square root of *N*. An SNR of 2.5 is obtained after averaging *N* = 1000, 1800, 7200 and 85 000 snapshot exposures for peaks at cosψ = 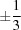, 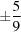, 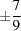 and ±0.4, respectively. While simulations of a simple twinning model (shown in Fig. 5 ▸ *b*) only reveal peaks at cosψ = 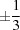, 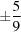 and 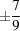, additional CXS peaks in the data with an SNR > 2.5 (Figs. 3 ▸ and 4 ▸) may indicate more complicated structures. Each measured CXS peak represents a potential constraint on atomic models, and these additional peaks could be used to refine more complicated twinning models. The ability of CXS to identify complex atomic-scale structures from solution data has potential for a wide range of applications, including structural studies of proteins where crystallography is not feasible.

## 4. Discussion   

### 4.1. The twinning signal   

We consider a twinned NP to be a single molecular unit with a unique orientation 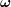. A twinned NP will have two or more crystal domains, which are identical in structure and related by a mirror reflection across a twinning plane. Here, we assume that the momentum transfer vectors of twinned domains are related by a rotation operator, **T**, corresponding to the twinning reflection in real space. Physically, this implies that the twinned domains will scatter correlated photons into different vectors of the same magnitude, with the constraint that the angle between these vectors is determined by **T**. The CXS information for a twinned NP is richer than that of a mono-domain NP because the operation **T** increases the number of possible momentum transfer differences between correlated photons scattered from a given twinned NP. In other words, the CXS signal arising from twinned NPs will contain angular correlation peaks in addition to those observed from mono-domain NPs (Fig. 5 ▸).

We assume each gold crystal domain has a well defined face-centered-cubic (f.c.c.) lattice structure. In this paper we only discuss correlations arising from the {111} family of planes. There are four distinct {111} planes: 111, 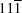, 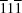 and 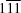, and the mirror-symmetric planes, 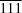, 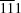 and 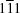, 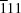. From an exposure of gold NPs in solution, photons scattered from these crystallographic planes give rise to a Bragg ring at *q*_111_ = 2π/*d*_111_, where *d*_111_ = 2.35 Å is the corresponding interplanar spacing. Notice how this Bragg ring appears as noise on a per-exposure basis (Fig. 2 ▸). Hidden beneath the noise level are correlated {111} photons, separated by specific angles in momentum space. We can predict these angles analytically for both mono-domain and twinned NPs. Let 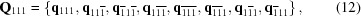
be the set of {111} Bragg vectors, each normalized to unity (|**q**| = 1), such that *e.g.* 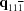 = (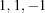)/3^1/2^. For a mono-domain NP, these are the possible directions where {111} photons will scatter. We can express analytically which cosines cosψ correspond to the angular differences between correlated photons by forming the sequence 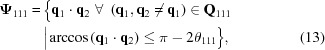
where the inequality is a result of the geometric constraint on ψ [equation (6)]. Evaluating the sequence 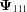, we find that it only contains values 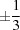. This is in agreement with the expected CXS signal for a mono-domain f.c.c. NP (Fig. 5 ▸ *a*).

As mentioned above and as indicated in our main result, the CXS information will be richer for multi-domain twinned NPs. Consider the following simple model for two f.c.c. tetrahedra joined by a twinning plane. Let each face of the tetrahedra be a {111} plane. When joined, the tetrahedra will have one plane in common, referred to as the twinning plane. The atomic coordinates of the twins are related to one another by a reflection about this plane. We refer to this twinned structure as a nearest-neighbor tetrahedron (NNT). Larger structures, *e.g.* decahedra and icosahedra, can be assembled with NNTs (Fig. 5 ▸ *b*). We call the twins twin_*A*_ and twin_*B*_. In this simple model, we let the twinning plane have Miller indices *h* = 1, *k* = 1, *l* = 1, and hence twin_*A*_ is oriented relative to twin_*B*_ *via* a rotation of π about the vector perpendicular to the (111) plane. This operation is given by the matrix 
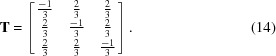
Let us define the set of momentum transfer vectors for the NNT model as 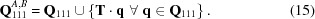
This new set of vectors reveals that the NNT structure can produce correlated photons whose angular differences are determined by the cosines 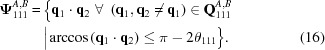
If π − 2θ_111_ > arccos(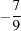), *i.e.* if the photon wavelength λ < 1.57 Å, then 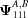 will only contain the values 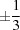, 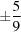 and 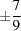 (Fig. 5 ▸ *b*). Indeed, our data show peaks at these angles, indicating the presence of twinning (Fig. 3 ▸). Note that the information content of CXS depends solely on the scattering factor of the individual molecule in solution. Depending on the growth process, gold NPs have been observed to grow into many complicated twinned shapes. In these so-called multiply twinned particles, there are additional correlations which can arise due to next-nearest-neighbor tetrahedra and so forth, as evident in our main result (Fig. 3 ▸ *b*).

### 4.2. CXS *versus* X-ray powder diffraction   

A powder pattern of twinned NPs will look identical to a powder pattern of non-twinned NPs. This is because a powder pattern measures the isotropically averaged scattering factor of the nanoparticles in solution [equation (S31) in the supporting information]. Since powder patterns are one-dimensional measurements in scattering angle, they cannot distinguish one twin domain from another. In contrast, CXS is a three-dimensional measurement [equation (S36) in the supporting information]. If one computes angular correlations of the intensities recorded in the diffraction pattern, peaks will emerge at specific angles [*e.g.* equations (13) and (16)], giving rise to a CXS signal that distinguishes twinned from non-twinned NPs.

### 4.3. Determination of biomolecular structure from solution measurement   

As emphasized by Z. Kam in his original 1977 paper, ‘…the method is particularly advantageous for structural determination of assemblies consisting of many macromolecules like viruses, ribosomes, and muscle filaments … and for obtaining structural information about membrane proteins *in situ*.’ (Kam, 1977 ▸). In the present paper we have now demonstrated the experimental capability of CXS for discerning complex molecular details on an atomic scale from true solution measurements. This establishes that the theoretical basis proposed by Kam can be applied to real samples containing a large number of molecules. The degree of averaging required to obtain a reasonable SNR at an atomic scale will certainly depend on the X-ray fluence and scattering power of the sample molecules. In this paper, we have taken advantage of the strong scattering cross section of gold to establish analysis techniques. Additional experimental work is needed in order to apply CXS to organic and biological molecules, where the scattering power is much lower per molecule. Methods for correcting for solvent scattering also need to be established. Contrary to our experiment, where solvent and sample scattering were physically separated in momentum space, the scattering from biomolecules will generally overlap with that from the solvent. The unique advantage for determination of biomolecular structure using CXS, compared with crystallography, NMR spectroscopy or electron cryomicroscopy, lies in its potential for taking snapshots of molecules in motion on XFEL-pulse time scales (tens of femtoseconds). For this reason, it is fair to say that the application of CXS to the study of time-delayed changes in biomolecular solution scattering in response to chemical or physical stimuli has the potential to greatly advance our understanding of the nature of biomolecular interactions.

## 5. Summary   

Advances in X-ray instrumentation and sources (*e.g.* in XFEL technology) have recently reached a critical point from which CXS has become feasible (Emma *et al.*, 2010 ▸; Ishikawa *et al.*, 2012 ▸). Consequently, the technique itself is still in its infancy. With our validation example, we have demonstrated that photon correlations from XFEL solution scattering can be used to reveal detailed information regarding the local molecular structure. We outline a method used to accumulate the correlations on a single-exposure basis, even in the presence of significant systematic noise (*e.g.* detector shadows), as well as noise arising from the innate randomness of molecular orientations in a solution sample. The true power of a CXS measurement is in the richness of its information. Here we have only reported the measurement of intensity auto-correlations at a single scattering vector magnitude, but even more information is contained in the cross-correlations and auto-correlations of all measured scattering vectors. As sample-injection and data-collection tools continue to improve, so should the ability to refine the angular intensity correlation functions hidden within solution scattering measurements, providing a means for better model fitting and a better understanding of molecular structure.

## Supplementary Material

Supplementary appendices and figures. DOI: 10.1107/S2052252516013956/cw5011sup1.pdf

## Appendix A Median absolute deviation filter steps

Given an observation *f*(*x*), *e.g.* an angular intensity, we can:

(i) Find the absolute deviation from the median of each observation *f*(*x*), *i.e.*

(ii) Set the modified standard score for each observation to be

(iii) Check whether *z*(*x*) is greater than some outlier threshold, ζ. For the purpose of separating the bright intensities from the moderate intensities, we let ζ = 2.5.

## Appendix B Estimating the size of NP scattering domains

### b1. Small twinned domains

From the Scherrer equation one can relate the size of a Bragg peak in reciprocal space to the size of the corresponding crystallographic domain in the NP. We define the NP size *s* as the cube root of the domain volume. By the Scherrer equation, we have

where *K* is a constant dependent on the shape of the domain, λ is the photon wavelength, β is the FWHM of the Bragg peak in radians and θ is half of the Bragg scattering angle at momentum transfer magnitude *q*:

For {111} planes in f.c.c. tetrahedral domains, *K* ≃ 0.89. Typically, a Bragg peak is modeled as the convolution of a Gaussian profile (the domain size) with a Lorentzian profile (the domain strain), otherwise known as Voigt profile. By fitting Voigt profiles to Bragg peaks, one can estimate β, and hence the size of the domain which scattered the Bragg peak photons.

In the case of CXS of small-domain NPs, our assumption is that a single exposure is too noisy to measure individual Bragg peaks. However, by averaging the correlations of many exposures, we can resolve correlated Bragg peaks (CXS peaks) which are also related to the size of the NP domains.

A CXS peak is the average self-convolution of all correlated Bragg peaks in each exposure. If we ignore strain contributions to the Bragg peak FWHM, β, then we can model the Bragg peak as just a Gaussian profile, and hence the CXS peak is a self-convolution of a Gaussian (note that the self-convolution of a Gaussian results in another Gaussian whose width is wider by a factor of 2^1/2^). With this, we define the FWHM of the CXS peak to be

We simulated CXS for a decahedron NP composed of five identical tetrahedral domains of side length *a*_sim_ ≃ 77.5 Å. We can compute *s*_sim_ directly as the cube root of the volume of one of the regular tetrahedra:

We can also evaluate *s*_sim_ using the Scherrer equation (19) combined with equation (21):

By fitting a Gaussian to a simulated CXS peak at cosψ = , we find the width δ_sim_ ≃ 0.055 rad (see Fig. S10 in the supporting information), hence *s*_sim_ ≃ 34.7 Å, in agreement with equation (22).

From the difference correlation of the moderate intensities, , we measure δ^m^ ≃ 0.032 rad, corresponding to a domain size of  ≃ 59.8 Å. For regular tetrahedral domains, this corresponds to a side length of

For a decahedral particle composed of five regular tetrahedra of side length *a* ^m^, the apparent diameter can be approximated as the circumradius of the pentagon whose side length is also *a* ^m^:

We conclude that this is an approximate lower bound on the diameter of the relatively small twinned NPs that we measured. We validate this conclusion with our examination of the bright Bragg spots in each snapshot exposure and the corresponding CXS peak width (section B2 below).

### b2. Large domains

On each image, there are Bragg rings from the gold NPs and, on the Bragg rings, there are bright Bragg spots which appear as outliers, defined in the main text as . Because the Bragg spots are above the noise, we can measure their width and hence gather information on the corresponding domain sizes. We construct a distribution of the Bragg spot widths by performing the following steps in order:

(i) Identify the bright Bragg spots on each Bragg ring image.

(ii) Measure the angular FWHM of the bright Bragg spots, β.

(iii) Repeat for many images to construct a histogram.

This distribution, *L*(β), gives the relative number of NP domains per exposure whose size corresponds to a Bragg spot of width β (Fig. S11 in the supporting information). The correlation of the bright Bragg spots, , does not show any strong signs of twinning (only having peaks at cosψ = ) and has peak width(s) δ^b^ ≃ 0.019 rad. [One can use the distribution *L*(β) to estimate δ^b^ directly; for details, see section B2.1 below].

A CXS peak width of δ^b^ = 0.019 rad corresponds to an NP domain side length (assuming tetrahedral domains) of

where we have made use of equations (23) and (24). Note that *a* ^b^ is smaller than the most commonly observed domain (which produced bright Bragg spots), whose corresponding side length we can calculate using the distribution of bright Bragg spots:

where

The fact that δ^b^/(2^1/2^) >  (or *a* ^b^ < ) indicates that the smaller domains in the distribution *L*(β) are spreading out the measured CXS peak. From these results, we conclude that the CXS peak width, δ, corresponds to an approximate lower bound on the NP domain size which contributed to . We expect these conclusions to hold for the distribution of small twinned NP domains.

#### b2.1. Using a distribution of Bragg peak widths to estimate a corresponding CXS peak width

Consider that the Bragg spots are Gaussians with FWHM β. Then, as mentioned in section B1 above, the correlation peak width is a convolution of two Gaussians, which is itself a Gaussian of width δ = 2^1/2^β. Keeping in mind that we have a distribution of NP sizes [corresponding to the distribution *L*(β)], we can model the FWHM of the outlier correlation peak (δ^b^) directly as the FWHM of the sum of Gaussians whose FWHM values are δ and whose amplitudes are *L*(β):

where σ_δ_ is the standard deviation of the convolved Gaussian whose FWHM is δ:

Note that the mean is not important in this calculation, which is why G_*L*_(ψ) has a mean of 0. Numerically, we find that the FWHM of G_*L*_(ψ) is roughly 0.017 rad, in good agreement with the measurement (0.019 rad).

## Appendix C Gaussian fitting to the difference correlation of the moderate intensities

After averaging all exposure difference correlations, we determined a set Γ of local maxima  in . Peaks were identified by first applying a Savitzky–Golay filter and a smoothing convolution to , and then searching for local extrema (see Fig. S7 in the supporting information).

Then, for each , we defined a Gaussian function

The offset *b* takes into account any residual background terms (*e.g.* the low-frequency background shown in Fig. S6 in the supporting information). The amplitude *A* is our measure of the CXS signal from the gold NPs (how far the CXS signal peaks above the background). The width η of the CXS peak is proportional to the size of the average NP domain which scattered the correlated photons (similar to how the Bragg peak width is proportional to the size of the NP domains).

By employing the Levenberg–Marquardt nonlinear least-squares algorithm, we obtained the fits  to each detected peak. With these fits, the total fitted CXS signal can be represented by a sum of Gaussians

Practically, we divided the detected maxima in Γ into ten subsets of neighboring local maxima, fitted partial sums to each subset and summed the results to achieve the fit (Fig. S8 in the supporting information).

## Appendix D Calculation of the signal-to-noise ratio (SNR)

We define the SNR of the CXS peaks indexed by γ to be

where *A*_γ_ is the amplitude of the CXS peak as measured from the noise level [the same *A*_γ_ that is defined in equation (32)] and σ is estimated to be the standard deviation of the inter-difference correlation, defined as

We compute  by randomly selecting pairs of exposures  and . If the exposures are paired in a way that minimizes artifactual variations (see section S1.7 in the supporting information), then the standard deviation of equation (34) is a good estimate of the theoretical noise σ associated with a CXS measurement. This technique for noise estimation is useful in situations where the CXS signal is continuous, *e.g.* in the case of soft-matter scattering or smaller NPs with broad Bragg reflections. Fig. S9 in the supporting information shows the scaling of *A*_γ_ and σ for the CXS peak in  at . The fitting of *A*_γ_ was a noisy process, especially for the lower values of *N* where the signal level is close to the noise level. We ran the fit multiple times until convergence of the amplitudes was reached.
