# Supplementary material for: Angular correlations of photons from solution diffraction at a free-electron laser encode molecular structure
Source: IUCrJ. 2016 Sep 26;3(Pt 6):420–9. doi: 10.1107/S2052252516013956 (PMC5094444; doi:10.1107/S2052252516013956)
Supplement: Supplementary file 1 [file m-03-00420-sup1.pdf]

IUCrJ

Volume 3 (2016)

Supporting information for article:

Angular correlations of photons from solution diffraction at a free-electron laser encode molecular structure

Derek Mendez, Herschel Watkins, Shenglan Qiao, Kevin S. Raines, Thomas J. Lane, Gundolf Schenk, Garrett Nelson, Ganesh Subramanian, Kensuke Tono, Yasumasa Joti, Makina Yabashi, Daniel Ratner and Sebastian Doniach

Appendix S1
Data Analysis

S1.1. Data acquisition

Scattered photons were recorded on an area detector perpendicular to the forward photon beam. The detector we used was the MPCCD octal sensor, consisting of 8 application-specific integrated circuits (ASICs) which are read out simultaneously, up to 60 Hz. The raw data

- are stored on an external user-restricted server, where they remain for one year before being moved to tape storage.
- are accessed using in-house SACLA data conversion programs, which output the data as user-accessible hdf5 files.

The in-house software has an option to store reconstructed images, which we employ. The ASICs are assembled into an approximate image, and the relative gains are adjusted. Each reconstructed MPCCD image has 2399×2399 pixels. The boundary of each image is padded with zeros (Fig. S1), so that the reconstructed image size doesn't change if the panels themselves are adjusted between experiments. Each pixel has an integer coordinate (p_x, p_y), which also serves as it's array index. We denote the measurement of each pixel during an exposure i as $I_i^{\mathrm{cart}}(p_x\,,\,p_y)$ where "cart" indicates the image is in cartesian coordinates.

S1.2. Polar interpolation of the pixel data

Our first task is performing a polar interpolation on the data

$$I_i^{\mathrm{cart}}(p_x\,,\,p_y) \rightarrow I_i(p_r \equiv r\,, p_\phi \equiv \phi) \tag{S1}$$

for a narrow range of $r \in [r_{\mathrm{low}}, r_{\mathrm{high}}]$ which encompasses the Bragg ring q_{111} (Fig. S1). This serves to reduce the effective size of the data, which is good for an analysis stream on a large dataset. Loading hundreds of thousands of large images in and out of random-access memory can slow down computation significantly. In this sense, it is better to work with a snippet of each image that we find interesting, which, in this case, is the region near the Bragg ring. The azimuthal pixel value is given by

$$\phi = \arctan\left(\frac{p_y - p_b}{p_x - p_a}\right) \tag{S2}$$

The point (p_a, p_b) is where the X-ray beam axis intersects the detector. We approximate that (p_a, p_b) remains constant throughout the experiment. One can extrapolate this point of intersection by using the Bragg rings themselves under the assumptions that they are circularly symmetric about the beam axis, and that the detector is perpendicular to the beam axis.

The radial pixel value r is related to the momentum transfer magnitude q via

$$r = \frac{d}{\Delta p}\ \tan\left(2\ \arcsin\left(\frac{q\,\lambda}{4\,\pi}\right)\right) \tag{S3}$$

where d, Δp, and λ are the sample-to-detector distance, square pixel length, and photon wavelength, respectively.

With these definitions, we use an elementary floor-nearest-neighbor interpolation to approximate the polar image:

$$I_i(r, \phi) = I_i^{\mathrm{cart}}\left(\lfloor r\ \cos(\phi) + p_a \rfloor, \lfloor r\ \sin(\phi) + p_b \rfloor\right) \tag{S4}$$

where $\lfloor\ \rfloor$ is the floor operation.

S1.3. Working with a pixel mask

Throughout our entire analysis, we work with masked images. We let $M(r\,,\phi)$ represent the polar image mask, a binary image used to exclude the gaps and boundaries surrounding the detector ASICS (panels). Masked pixel values are 0 and usable pixel values are 1. Typically there is a mask M^{cart} for the cartesian images. In this case we would define the polar mask to be

$$M(\,r\,,\phi) = M^{\text{cart}}\left(\lfloor\, r\,\cos(\phi) + p_a\rfloor, \lfloor\, r\,\sin(\phi) + p_b\rfloor\right) \tag{S5}$$

Commonly, large scale sensors like these have signal spikes near the edges, so the ASIC edges should be included in the mask. As an example, the mean signal in a masked polar image is defined as

$$\overline{I}_i = \frac{\left(\sum\limits_{r\,,\phi} M(r\,,\phi)\; I_i(r\,,\phi)\right)}{\left(\sum\limits_{r\,,\phi} M(r\,,\phi)\right)} \tag{S6}$$

For our experiment, we used a fixed mask M^{cart} and hence M for all images, but it can also vary throughout a given experiment.

S1.4. The radial position of the 111 Bragg ring

In this particular experiment, the sample jet was unstable. Consequently, the sample-to-detector distance fluctuated on a shot by shot basis. In extreme cases, the viscous lipid-cubic-phase solution would kink and clog around the syringe needle tip, causing significant fluctuations in r_i^* (Fig. S3).

We will denote the $\{111\}$ Bragg ring radial position in exposure i by r_i^*, i.e. the radial pixel ring corresponding to q_{111}. Since we do not know the precise sample-to-detector distance of each exposure i, we estimate

$$r_i^* = \mathrm{argmax}\left[\langle I_i\,(\,r\,,\phi)\,\rangle_\phi\right] \tag{S7}$$

where $\langle \ldots \rangle_\phi$ denotes the discrete average over ϕ. Therefore, r_i^* corresponds to the angle where the radial profile of the polar image is maximum. Because gold is the only sample component which scatters at these high angles, we assume this is a robust approach.

S1.5. The angular intensity profile $I_i(\phi)$ along the Bragg ring

For the purpose of this paper, we found it sufficient to represent the Bragg ring profile by

$$I_i(\phi) = I_i(r = r_i^*, \phi) \tag{S8}$$

We sample ϕ at N_ϕ evenly spaced points along the Bragg ring. We fix $N_\phi \geq 2\,\pi\,r_{\mathrm{high}}$ where r_{high} is shown in Fig. S1. In this way we will sample azimuthally at unit pixel precision at the highest anticipated Bragg ring position (r_{high}).

S1.6. Quantifying angular anisotropies

Shadows, beam polarization, and sample inhomogeneity, are but a few sources of systematic noise which can give rise to large angular anisotropies in $I_i(\phi)$. One can see these by eye (Figs. S1, S4). We have a method for overcoming these effects, which depends on the pairing of exposures with similar anisotropies. Here we discuss the quantification of the angular anisotropy.

In order to quantify the anisotropy, we fit 15th degree Chebyshev polynomials of the first kind to the angular intensity profile $I_i(\phi)$. Chebyshev polynomials of the first kind are defined by

$$y(\phi) = c_0\,T_0(\phi) + c_1\,T_1(\phi) + \cdots + c_{15}\,T_{15}(\phi) \tag{S9}$$

where

$$\begin{aligned} T_0(\phi) &= 1 \\ T_1(\phi) &= x \\ T_{n+1}(\phi) &= 2\,x\,T_n(\phi) - T_{n-1}(\phi) \end{aligned}$$

The fit is a simple least squares which minimizes the residual

$$E_i = \sum_{\phi} \left| \, I_i(\phi) - y_i(\phi) \right|^2 \tag{S10}$$

Note in Fig. S4 the large Bragg spots (peaks). These large signal spikes will bias the residual E_i, hence we mask them prior to fitting the polynomial. To detect the signal spikes we use the median outlier filter described in appendix A. We let y_i^* be the Chebyshev polynomial which minimizes the unbiased residual. Figure S4 shows two polynomial fits, one to the raw data and another to the data without the Bragg spots. The pairing of exposures according to their angular anisotropies is critical for our analysis, as detailed in the following sections.

S1.7. Exposure pairing

For our reported results, we made use of the difference correlation, which involves subtracting pairs of exposures and correlating the residuals. Our data were divided up into 85 experimental runs, and each run represented an average of 4500 usable exposures. We considered a usable exposure to be one where the X-ray shutter was open and the X-ray laser was operating properly (occasionally the laser pulses would cease during a run from complications upstream). We acquired roughly 3.8×10^5 usable exposures, and we did not attempt to compare each exposure with every other exposure. Rather, we only compared and paired exposures that occurred during the same experimental run.

We began by selecting all exposures for analysis according to their total average signal $\overline{I}_i$ as defined in equation (S6) (Fig. S5). We ignored exposures that were too weak ($\overline{I}_i < 300\,\text{counts}$) because it is likely that they were recorded when the sample injector failed. Similarly, we rejected exposures that were too strong ($\overline{I}_i > 3000\,\text{counts}$), for they may include non-linear effects on the detector such as faulty pixel responses.

After filtering based on mean intensity, exposures within a certain run were grouped according to their respective r_i^*. Each exposure i was assigned to a subgroup based on the floored value $\lfloor r_i^* \rfloor$, i.e. the closest integer less than r_i^* (the vertical orange lines in Fig. S3 represent the group bins). Pairs were constrained to be formed using exposures from the same subgroup. The pairing process involved an optimization step in which exposures were recursively compared to each other; forming subgroups for pairing serves to reduce the required computation time.

We required that an exposure can only be used once during analysis. Each exposure i was paired with an exposure j according to their azimuthal anisotropies, quantified by the fitted polynomials y_i^*, y_j^* (azimuthal anisotropies should be similar for similar positions r on the polar image; the subgrouping described above is advantageous in this regard). We used the squared Euclidean distance

$$\epsilon_{i,j} = \sum_{\phi} \left(y_i^*(\phi) - y_j^*(\phi) \right)^2 \tag{S11}$$

as a metric of comparison between two exposures. Let $\mathcal{P}$ represent a set of pairings in which each exposure is paired, and no exposure is paired twice (it is understood that if there is an odd number of exposures in a subgroup, then a single shot will remain unpaired and thus not used in the analysis). We can define the total distance between paired exposures as

$$d = \sum_{i,j \in \mathcal{P}} \epsilon_{i,j} \tag{S12}$$

A good pairing seeks to minimize d. This is a computationally hard problem, therefore we approximate an optimal pairing $\mathcal{P}$.

S1.8. Computing the difference intensity profile

The difference intensity profile is defined as

$$I_{i,j}(\phi) = \widehat{I}_i(\phi) - \widehat{I}_j(\phi) \tag{S13}$$

where we define the normalized angular intensity profile

$$\widehat{I}_i(\phi) = I_i(\phi) \left(\frac{\sum_\phi M_i(\phi)}{\sum_\phi M_i(\phi)\, I_i(\phi)} \right) \tag{S14}$$

is the normalized intensity profile. We normalize prior to subtraction, otherwise the difference profile will be offset about zero, which will bias the correlation computation.

We combine the angular profile masks (which mask detector panel gaps, moderate/bright intensities, etc.) as

$$M_{i,j}(\phi) \;=\; M_i(\phi)\; M_j(\phi) \tag{S15}$$

S1.9. Computing the difference correlation

Now we discuss the actual correlation computation. Typically it should be straightforward, but we are correlating masked functions, and proper handling of the mask is essential. For each correlation angle Δ, we must keep track of the number of non-masked ϕ pairs, e.g.

$$N_{i,j}(\Delta) = \sum_\phi M_{i,j}(\phi)\; M_{i,j}(\phi + \Delta) \tag{S16}$$

The masked difference correlation for exposures i, j is then given by

$$D_{i,j}(\Delta) = \frac{\sum_{\phi} I^*_{i,j}(\phi)\; I^*_{i,j}(\phi+\Delta)}{N_{i,j}(\Delta)} \tag{S17}$$

where we define the masked difference profile

$$I^*_{i,j}(\phi) \equiv I_{i,j}(\phi)\, M_{i,j}(\phi) \tag{S18}$$

The computation time for computing equation (S17) scales as $(N_\phi)^2$ where N_ϕ is the number of sampled intensity values around the Bragg ring. Because $I_{i,j}(\phi)$ is periodic in 2π, we can employ a discrete fast-Fourier transform in order to speed up the computation of $D_{i,j}(\Delta)$. Let $A_{i,j}(k)$ be the discrete Fourier transform of the angular difference intensity profile

$$A_{i,j}(k) = \sum_{\phi} I^*_{i,j}(\phi)\; e^{-2\pi\,\imath\,\phi\,k\,/\,N_\phi} \tag{S19}$$

(where the symbol $\imath = \sqrt{-1}$). Let $B_{i,j}(k) = |A_{i,j}(k)|$ be the complex modulus of $A_{i,j}(k)$. By the Wiener-Khinchin theorem, the difference correlation is the real-valued inverse Fourier transform of $(B_{i,j}(k))^2$:

$$D_{i,j}(\Delta) = \Re\left[\; \frac{1}{N_\phi} \sum_{k} (B_{i,j}(k))^2\; e^{2\pi\,\imath\,k\,\Delta\,/\,N_\phi}\;\right] \tag{S20}$$

where $\Re\,[\ldots]$ ensures real-only output. Therefore, we can speed up the correlation computation time by computing fast-Fourier transforms.

Because the average value of a difference intensity profile $I^*_{i,j}(\phi)$ is 0, one can compute the Fourier transform on the full uniform domain for ϕ, i.e. for

$$\phi \in \left\{0\,,\, \frac{2\,\pi}{N_\phi}\,,\, \frac{4\,\pi}{N_\phi}\,, \ldots\, \frac{2\,\pi\,(N_\phi-1)}{N_\phi}\right\} \tag{S21}$$

This works because the masked values are also defined to be 0, so we are effectively replacing the masked ϕ components with the average value. Otherwise, one will need to compute and normalize by equation (S16) for each correlation, adding significant computation time.

S1.10. Code availability

The data analysis code is freely available through GitHub. Please email the corresponding author for details.

Appendix S2
CXS simulation

We define a solution as a set of identical, non-interacting objects (e.g. molecules) m, each with an independent orientation $\boldsymbol{\omega}$ relative to the X-ray beam axis, governed by the object's diffusion constant. We consider m as a collection of N_a atoms each with position vector

$$\boldsymbol{r}_j^m(t) = \boldsymbol{R}_\omega^m(t) \cdot \boldsymbol{r}_j + \boldsymbol{T}^m(t) \qquad 1 \leq j \leq N_a \tag{S22}$$

where $\boldsymbol{R}_\omega^m(t)$ is a rotation operator, $\boldsymbol{T}^m(t)$ is a translation operator representing the center of mass position of m at time t, and $\boldsymbol{r}_j$ is the position of the jth atom at an arbitrarily defined initial orientation. If we freeze the solution at an instant in time and expose it to X-ray photons of wavelength λ, then we can measure the scattering factor function

$$S(\boldsymbol{q}, t) = \left| \sum_m^{N_m} \sum_j^{N_a} f_j(q)\, e^{-i\,\boldsymbol{q}\cdot\boldsymbol{r}_j^m(t)} \right|^2 \tag{S23}$$

Here $f_j(q)$ is the atomic form factor of the jth atom, $\boldsymbol{q}$ represents a position in reciprocal space (e.g. of a pixel) at scattering angle

$$\theta = \arcsin\left(\frac{\lambda\, q}{4\pi}\right) \tag{S24}$$

and the outer sum is over all N_m exposed objects. In general, $S(\boldsymbol{q}, t)$ may be written as

$$S(\boldsymbol{q}, t) = \sum_{m}^{N_m} |A_\omega^m(\boldsymbol{q}, t)|^2 + \sum_{m \neq m'} A_\omega^m(\boldsymbol{q}, t) \left(A_\omega^{m'}(\boldsymbol{q}, t)\right)^* \tag{S25}$$

where

$$A_\omega^m(\boldsymbol{q}, t) = \sum_{j}^{N_a} f_j(q) e^{-i\, \boldsymbol{q} \cdot \boldsymbol{r}_j^m(t)} \tag{S26}$$

The strength of the interference term on the RHS of equation (S25) depends on both the concentration of the sample and the magnitude of the momentum transfer vector q. For large momentum transfer (wide scattering angles) and/or more dilute samples, the factors $e^{-i\boldsymbol{q}\cdot(\boldsymbol{T}^m - \boldsymbol{T}^{m'})}$ will approach zero, and we can neglect the second sum on the RHS such that we have

$$\begin{aligned}
S(\boldsymbol{q}, t) &= \sum_{m}^{N_m} |A_\omega^m(\boldsymbol{q}, t)|^2 && \text{(S27)} \\
&= \sum_{m}^{N_m} \left| \sum_{j}^{N_a} f_j(q)\, e^{-i\, \boldsymbol{q} \cdot \boldsymbol{r}_j^m(t)} \right|^2 && \text{(S28)} \\
&= \sum_{m}^{N_m} \left| \sum_{j}^{N_a} f_j(q)\, e^{-i\, \boldsymbol{q} \cdot (\boldsymbol{R}_\omega^m(t) \cdot \boldsymbol{r}_j + \boldsymbol{T}^m(t))} \right|^2 && \text{(S29)} \\
&= \sum_{m}^{N_m} \left| \sum_{j}^{N_a} f_j(q)\, e^{-i\, \boldsymbol{q} \cdot \boldsymbol{R}_\omega^m(t) \cdot \boldsymbol{r}_j} \right|^2 && \text{(S30)}
\end{aligned}$$

Therefore the measured scattering factor for a dilute solution is simply a superposition of single molecule scattering factors at various orientations $\boldsymbol{\omega}$. Instead of considering the precise time dependence of each object (molecule) in solution, however, we consider that, on average, each orientation $\boldsymbol{\omega}$ is occupied by a fixed number

of molecules $N_\omega = (N_m / \int d\boldsymbol{\omega})$, and that, at each instant, this number is fluctuating by some small amount $\alpha(\boldsymbol{\omega}, t)$ (we are borrowing notation directly from the original CXS paper by Zvi Kam in 1977). Consider the average isotropic scattering factor over all molecules

$$S(q) = N_\omega \int S(\boldsymbol{q}, \boldsymbol{\omega}) d\boldsymbol{\omega} \tag{S31}$$

where

$$S(\boldsymbol{q}, \boldsymbol{\omega}) = \left| \sum_j^{N_a} f_j(q)\, e^{-i\, \boldsymbol{q} \cdot \boldsymbol{R}_\omega \cdot \boldsymbol{r}_j} \right|^2 \tag{S32}$$

is the scattering factor of a molecule at orientation $\boldsymbol{\omega}$. Then we can represent $S(\boldsymbol{q}, t)$ as

$$S(\boldsymbol{q}, t) = S(q) + \int S(\boldsymbol{q}, \boldsymbol{\omega}) \alpha(\boldsymbol{\omega}, t) d\boldsymbol{\omega} \tag{S33}$$

Zvi Kam's main statement is that by measuring

$$\langle S(\boldsymbol{q}_1, t) S(\boldsymbol{q}_2, t) \rangle_t - S(q_1) S(q_2) \tag{S34}$$

one would resolve a correlation function

$$C(\boldsymbol{q}_1, \boldsymbol{q}_2) = N_\omega \int S(\boldsymbol{q}_1, \boldsymbol{\omega}) S(\boldsymbol{q}_2, \boldsymbol{\omega}) d\boldsymbol{\omega} \tag{S35}$$

or

$$C(q_1, q_2, \cos\psi) \propto \int S(\boldsymbol{q}_1, \boldsymbol{\omega}) S(\boldsymbol{q}_2, \boldsymbol{\omega}) d\boldsymbol{\omega} \tag{S36}$$

which depends only on the single particle scattering factor. The scattering factor in equation (S32) is what we simulate, given an arrangement of atoms. We can then easily calculate the expected CXS signal by evaluating the integral in equation (S36).

Appendix S3
List of supplementary figures

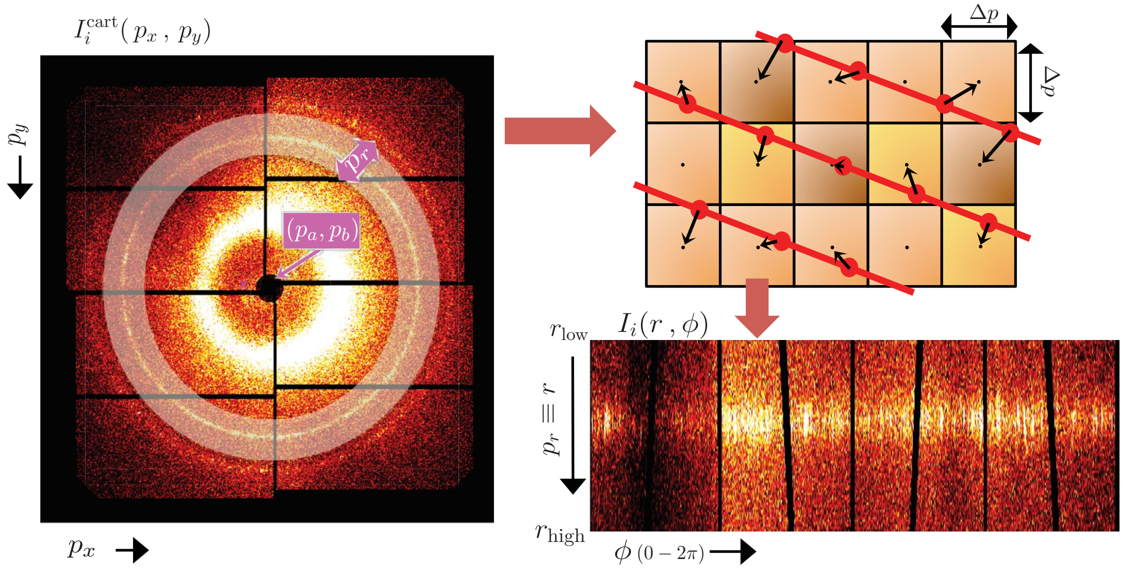

Fig. S1. A reconstructed scattering image of gold NPs, and the result of a floor-nearest-neighbor interpolation across the q_{111} Bragg ring. The polar image shown has the same resolution as the cartesian image. There appears to be a shadow on the image, which will lead to artifactual CXS signals.

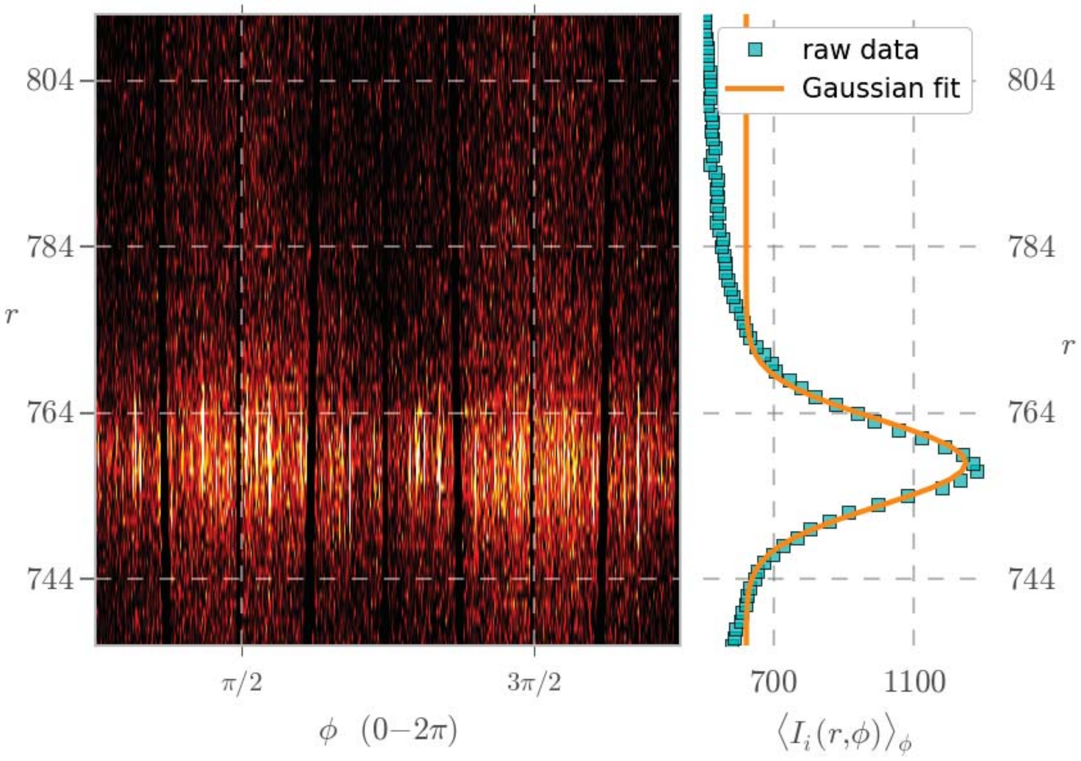

Fig. S2. *(Left)* A polar image $I_i(r, \phi)$ representing a single snapshot of the gold nanoparticles. *(Right)* The azimuthally-averaged intensity and a corresponding Gaussian fit. The center of the Gaussian corresponds to the {111} Bragg ring radial position, r_i^*

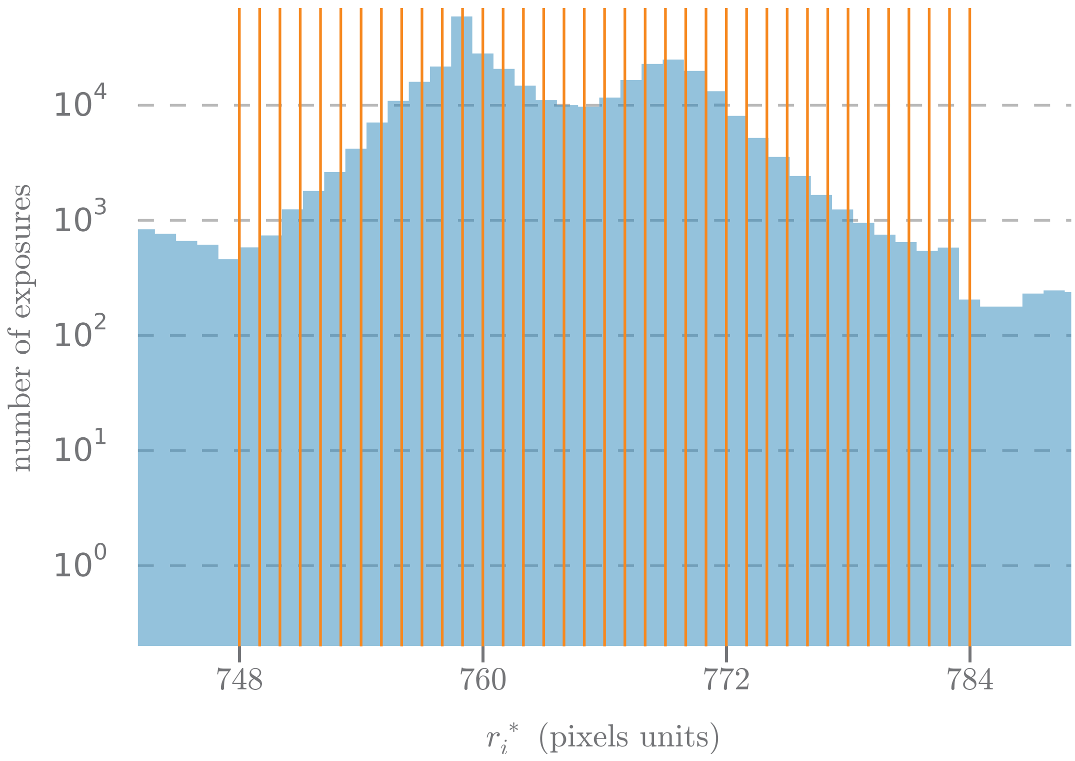

Fig. S3. A histogram of the radial pixel ring corresponding to q_{111}. We predict that the shape of this histogram (with the two peaks near 760 and 772) arises due to fluctuations in the injector system leading to different sample-to-detector positions. One way to fix this would be to use a gas focuses sample injector. Exposure pairing was a critical part of our analysis. We only paired exposures whose r_i^* were in the same radial bin, marked here by the orange vertical lines.

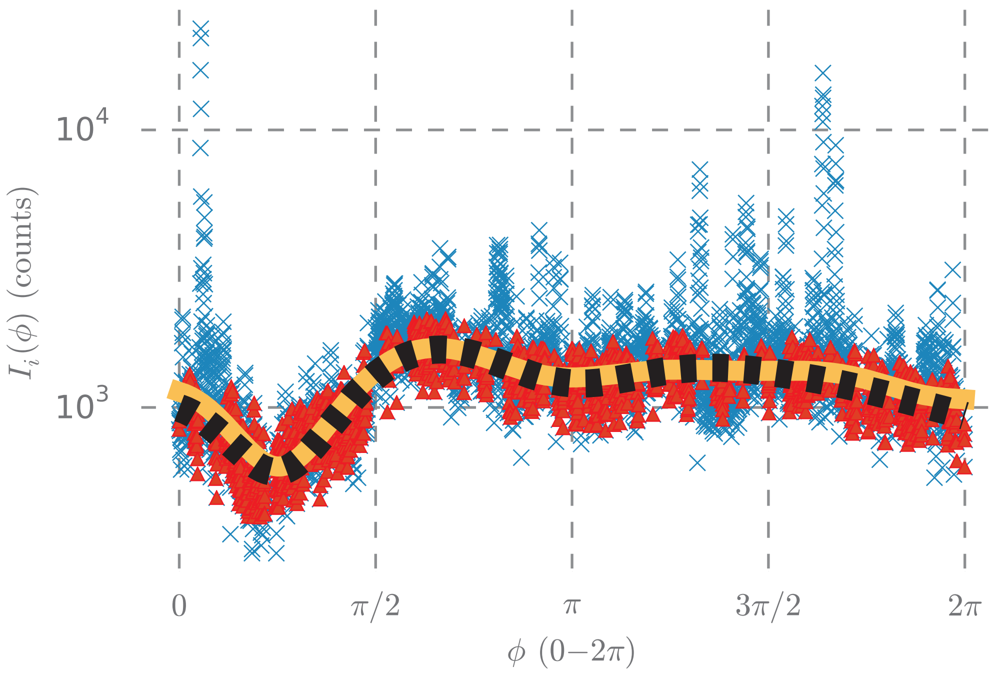

Fig. S4. In blue is the raw calculation of $I_i(\phi)$ for a single snapshot, which shows sharp peaks indicative of larger crystallite domains. Our motivation is actually to study less crystalline materials in the soft-matter regime, so we attempt to separate all signal associated with these larger, more crystalline nanoparticles. The solid yellow line is a biased polynomial fit to the raw data with the signal spikes (blue x markers). Shown in dashed-black is the unbiased polynomial, $y_i^*(\phi)$, fit to the data without the Bragg peaks (red triangle markers).

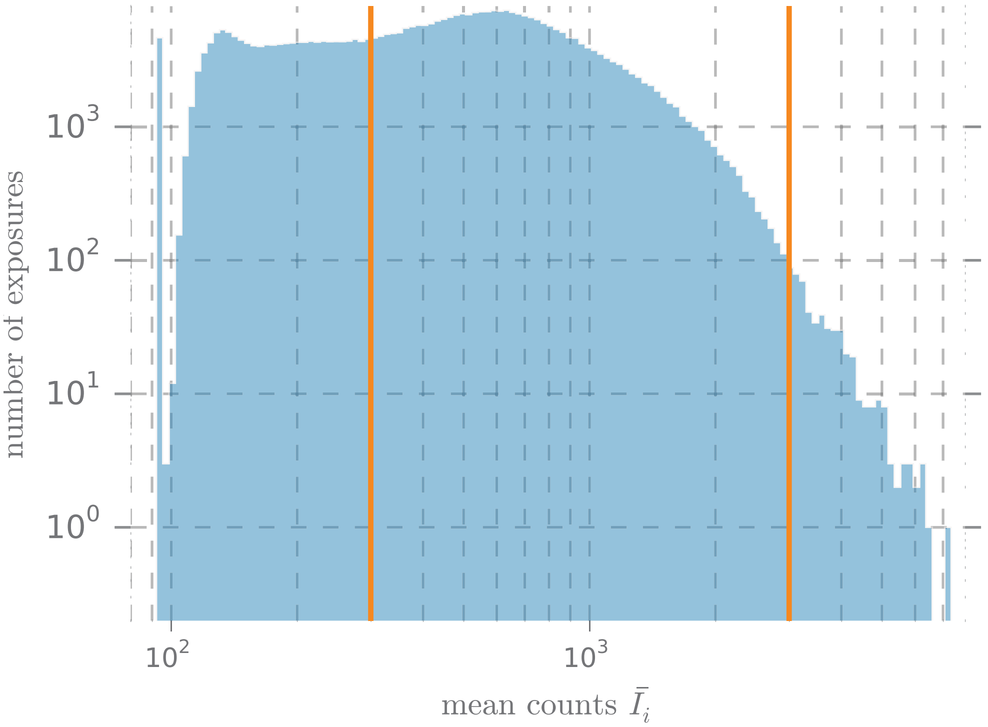

Fig. S5. A histogram of the mean intensity of every polar image $I_i(r, \phi)$ across all experimental runs. Only exposures whose mean intensity was greater than 300 units and less than 3000 units were analyzed ($300 \leq \overline{I}_i \leq 3000$).

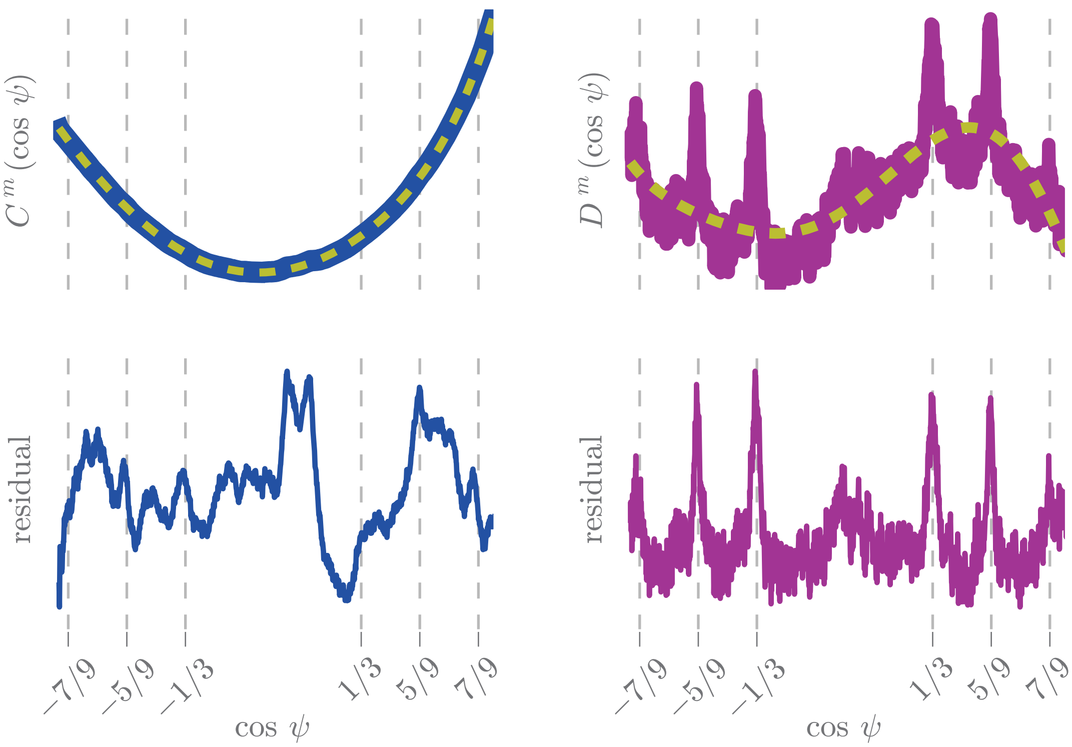

Fig. S6. Comparing the raw correlation of the moderate intensities $C^m(\cos\psi)$ to the difference correlation of the moderate intensities $D^m(\cos\psi)$. We fit 6th degree polynomials (dashed yellow) to the data and subtracted them to emphasize that the raw correlations contains signals which are certainly artifactual. These data represent averages over tens of thousands of exposures. Expected CXS signals for gold NPs are marked on the axis and shown with grid lines. Apparent in the figure, the difference correlation is a critical step in the analysis. Without it we would not be able to distinguish the gold NP CXS signal from the artifactual CXS signal. Low-frequency variation in the difference correlation (top-right) persists, and is due to extreme detector artifacts.

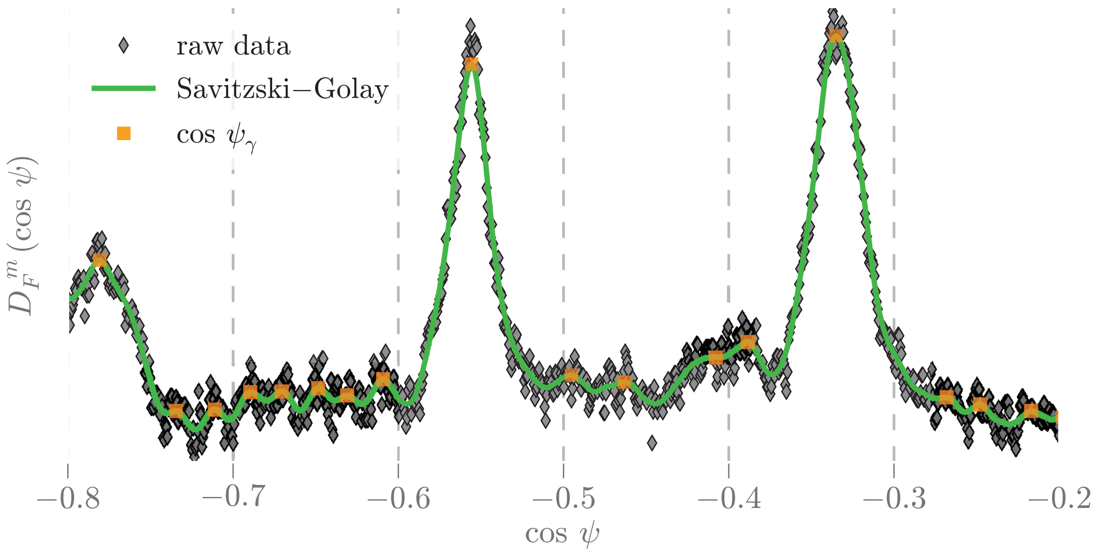

Fig. S7. A portion of the angular difference correlation. Smoothing is applied, and then peaks are located by calculating local extrema.

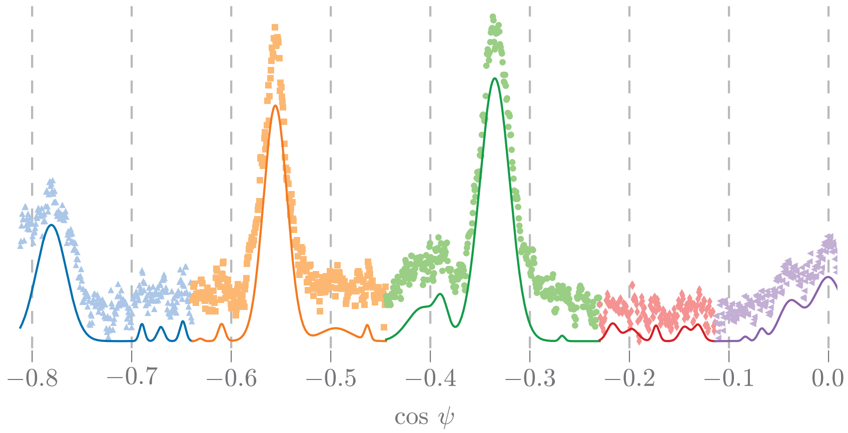

Fig. S8. The symmetric difference correlation $D_F(\cos\psi)$ and partial Gaussian fits $G(\cos\psi)$ as defined in equation (31). The different markers (triangle, square, circle, etc) represent different ranges over which the sum of Gaussians was fit.

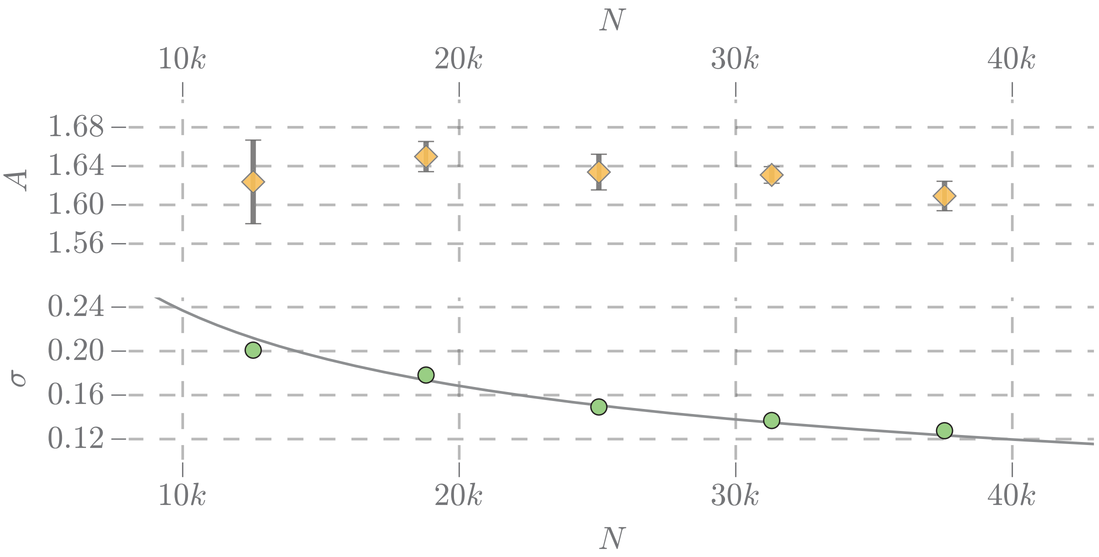

Fig. S9. *(Top)* The scaling and convergence of the Gaussian amplitude A_γ for the CXS peak at $\cos\psi = 1/3$. The error bar is one standard deviation across 200 fit attempts. *(Bottom)* The scaling of the CXS noise σ. The fitted curve scales as $N^{-1/2}$.

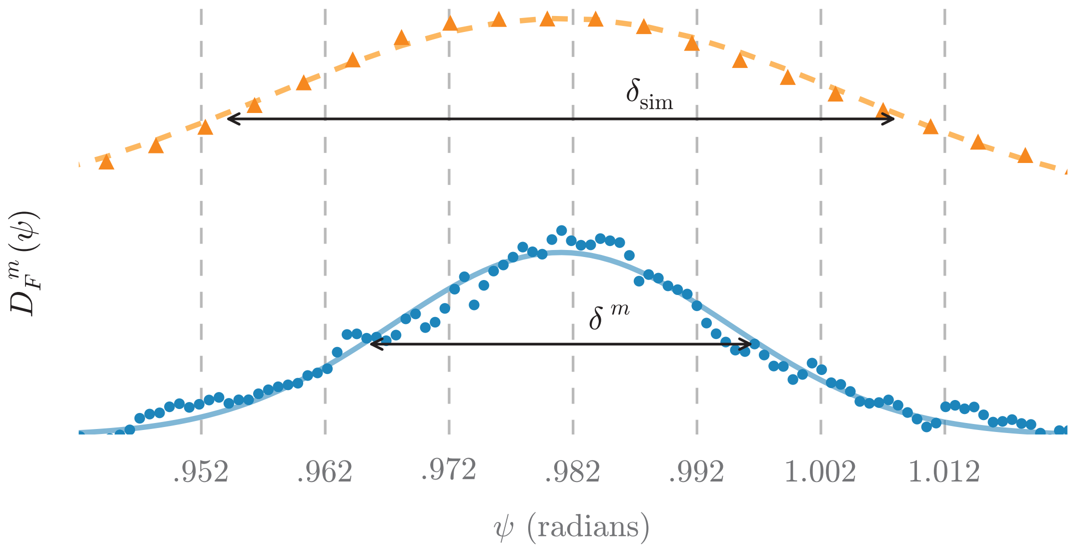

Fig. S10. Comparing CXS peak widths at $\cos\psi = 5/9$. *(Top)* The simulated CXS for a gold decahedron composed of five regular tetrahedrons of side length $a = 77.5$ Å (triangle marker). The FWHM, $\delta_{\rm sim}$, corresponds to an NP domain of size $s = 34.7$ Å. The dashed line is a Gaussian fit. *(Bottom)* The same CXS peak observed in the moderate intensity correlation, $D_F^m(\psi)$ (circle marker). The FWHM, δ^m, corresponds to an NP domain of size $s = 59.8$ Å. The solid line is a Gaussian fit.

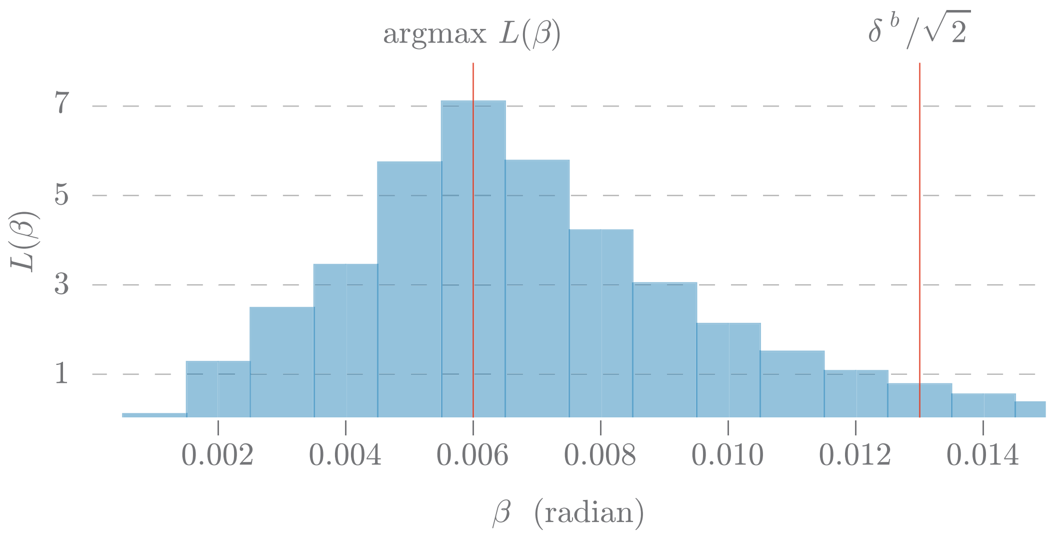

Fig. S11. The distribution $L(\beta)$ of FWHM values for the bright Bragg spots measured during a single snapshot exposure. This represents the relative number of NP domains whose domain size corresponds to a FWHM of β. The bright Bragg spots are a result of the large NP domains in the sample, and the average large-domain size is the peak in this histogram, denoted by argmax$L(\beta)$. If we assume the domains are tetrahedral, this would correspond to a domain whose side length is 46 nm. We note that these larger domains do not show significant signs of twinning in the correlation $D_F^b(\cos\psi)$.
